# Early cardiac rehabilitation after heart transplantation in a patient with limb-girdle muscular dystrophy: A case report

**DOI:** 10.1097/MD.0000000000029180

**Published:** 2022-07-29

**Authors:** Youngmo Kim, Min Kyung Park, Myung-Jun Shin, Yong Beom Shin, Hye Won Lee, Ra Yu Yun, Byeong-Ju Lee

**Affiliations:** a Department of Rehabilitation Medicine, Biomedical Research Institute, Pusan National University Hospital, Busan, South Korea; b Department of Rehabilitation Medicine, Biomedical Research Institute, Pusan National University Hospital and Pusan National University School of Medicine, Busan, South Korea; c Division of Cardiology, Department of Internal Medicine, Medical Research Institute, Pusan National University Hospital, Busan, South Korea; d Department of Rehabilitation Medicine, Rehabilitation Hospital, Pusan National University Yangsan Hospital, South Korea.

**Keywords:** cardiac rehabilitation, heart transplantation, muscular dystrophy

## Abstract

**Rationale:**

Cardiac rehabilitation (CR) after heart transplantation (HT) decreases the mortality rate and increases exercise capacity of patients. Dilated cardiomyopathy develops in most patients with muscular dystrophy (MD), leading to advanced heart failure, necessitating the use of left ventricular assist devices or HT. As the clinical outcomes of left ventricular assist devices and HT in patients with myopathy differ from those in patients without myopathy, CR adapted to patients with MD should be considered.

**Patient concerns:**

A 39-year-old man with limb-girdle muscular dystrophy developed dilated cardiomyopathy and underwent HT.

**Diagnosis:**

The patient was diagnosed as having limb-girdle muscular dystrophy in 1997.

**Intervention:**

Early CR was performed based on the patient’s physical condition and ability.

**Outcomes:**

With chest physiology, aerobic, and resistance exercises, the patient was able to walk using a walker 28 days after HT. This is important because his lower-extremity strength and walking ability were, to some extent, maintained after surgery.

**Lessons:**

Since an increasing number of patients with MD are undergoing HT, specific CR programs for these patients should be discussed.

## 1. Introduction

It is well known that cardiac rehabilitation (CR) after heart transplantation (HT) decreases the mortality rate and improves the exercise capacity of patients. As dilated cardiomyopathy develops in most patients with muscular dystrophy (MD), leading to advanced heart failure, left ventricular assist devices (LVADs), and HT are often required. Early CR is essential to prevent postoperative complications arising from bed rest and improve prognosis after HT. There is a consensus that a tailored program should be implemented, but there are no detailed guidelines for the rehabilitation of patients with MD. A patient with Becker’s MD reportedly received phase II CR,^[1]^ but no studies have reported a specific CR for patients with MD in the acute phase after HT. Patients with MD often support their upper body to compensate for weak lower-extremity muscle strength; therefore, sternotomy precautions (not using the upper extremity after median sternotomy) should be considered to avoid postoperative wound dehiscence. As the transplanted heart is denervated by the autonomic nervous system, the rating of perceived exertion (RPE), instead of the heart rate (HR), was used to determine exercise intensity.

Herein, we report a case in which these limitations were overcome using a specific phase I CR program in a patient with limb-girdle muscular dystrophy (LGMD).

## 2. Case report

A 39-year-old man was admitted to the cardiology department in December 2016 because of dyspnea. At 21 years of age, he was unable to run or ascend stairs because of muscular weakness. Blood tests, electromyography, and muscle biopsy were performed and the patient was diagnosed with LGMD in 1997. He has been walking with the support of a cane since 2005. In 2012, he was diagnosed with dilated cardiomyopathy accompanied by LGMD. He was admitted because of aggravation of heart failure eight times in the last five years. He underwent HT on February 1, 2017. He had a medical history of pulmonary thromboembolism and pneumonia, but no relevant family history.

**Figure 1.**
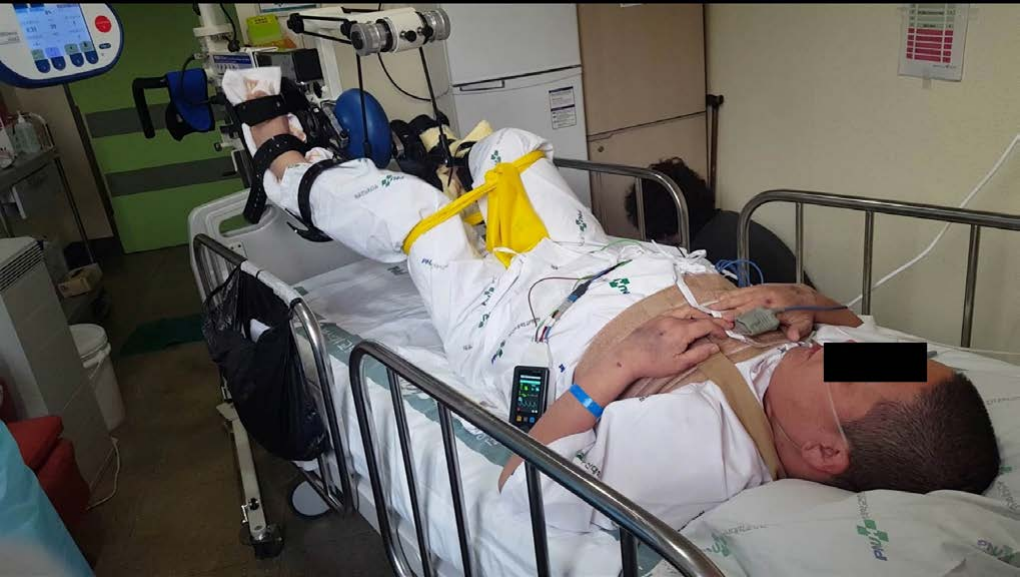
Exercise in an isolated room with a portable lower-body ergometer.

**Figure 2.**
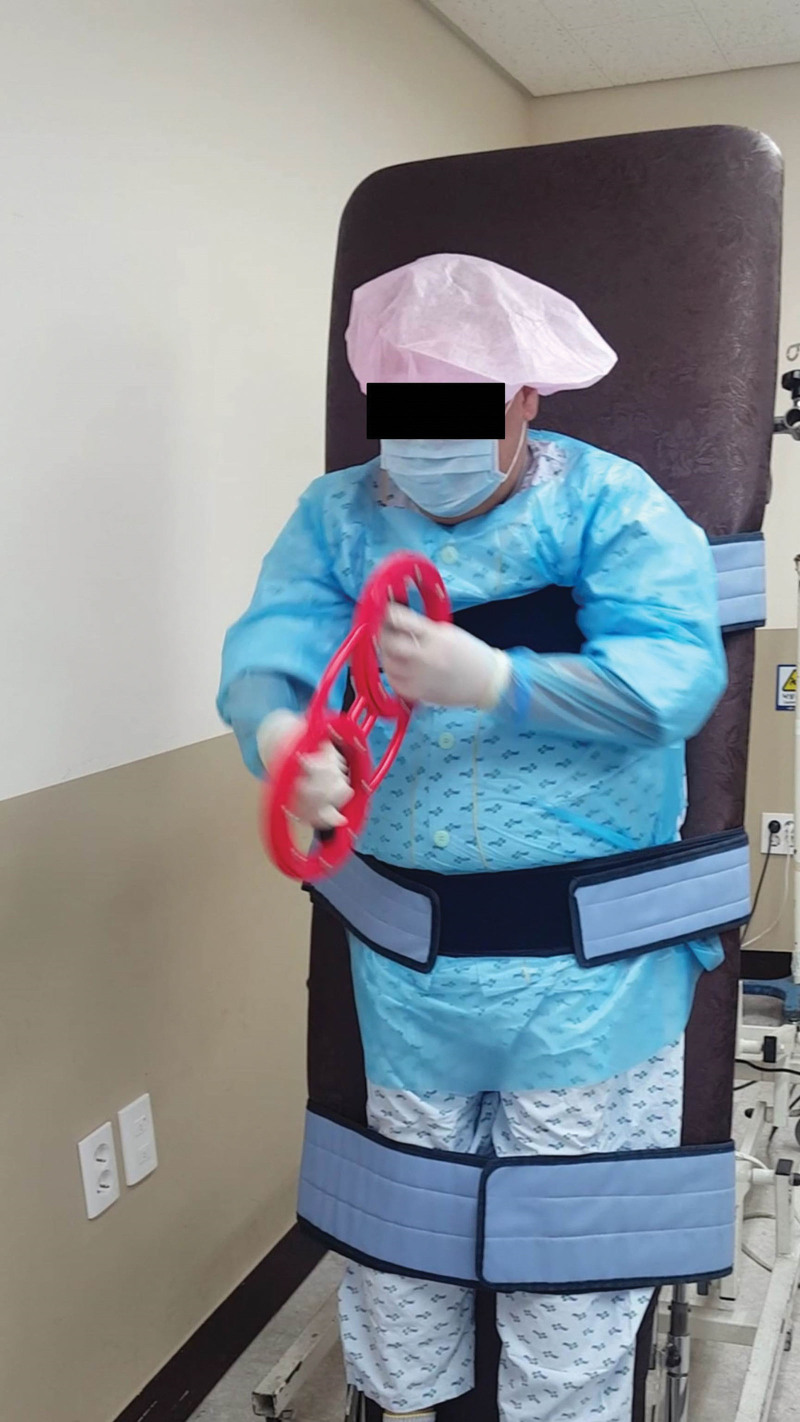
Exercise in an isolated rehabilitation room, standing on a tilt table with a portable hand-held ergometer.

**Figure 3.**
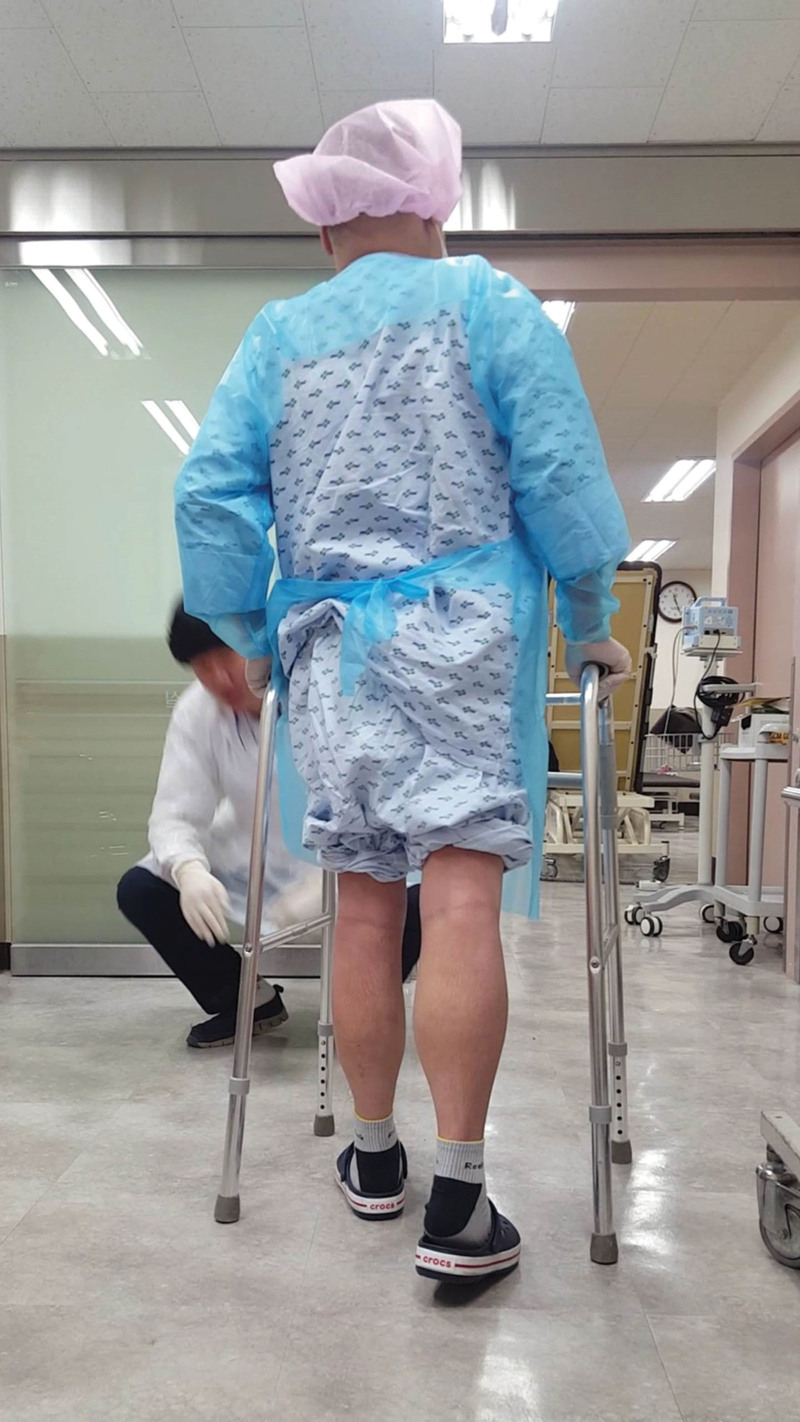
Walker gait on postoperative day 28.

On the first postoperative day, the patient was referred to the rehabilitation department and phase I CR was initiated. He was alert and could obey 3-step verbal commands. His muscle strength was as follows: good grade in the upper extremities; poor grade in hip flexors, extensors, abductors, and adductors; poor+ grade in knee flexors and extensors; and fair grade in ankle dorsiflexors and plantar flexors. He underwent chest physiotherapy, including mechanical insufflation-exsufflation, incentive spirometry, and cough splinting for 2 weeks in the intensive care unit. After 14 days of HT, the patient was transferred to the general ward under protective isolation. Muscle power in the lower extremities was extremely low when performing conventional CR. Moreover, he could not use a walking aid because of midline sternotomy. A portable half-automated ergometer (MOTOmed, RECK-Technik GmbH & Co. KG, Betzenweiler, Germany) was used for aerobic exercises with the lower extremities on the bed (Fig. 1). A portable hand-held ergometer (Burn Machine SB-4, Burn Machine, United States) was used, with both upper extremities held close to the torso and in accordance with sternotomy precautions (Fig. 2). The resting HR was 102 beats per minute and hardly changed during the exercise. Exercise intensity was adjusted to maintain the patient between 11 (fairly light) and 13 (somewhat hard) points on the 6–20 RPE scale. The exercise duration was 10 minutes. After 21 days of HT in an isolated protective rehabilitation room, the patient continued to exercise with a burn machine while standing on a tilt table (Fig. 2). The duration of the exercise was 25 minutes, response to HR remained blunt, and HR was elevated by 10 beats per minute and recovered 15 minutes after the end of the exercise. Twenty-eight days after CR, the patient was able to stand and walk with a walker (Fig. 3). After completing the rehabilitation program in each period (Table 1), the patient did not experience motor weakness in his lower extremities compared with the pre-HT motor weakness. Six months postoperatively, the sternotomy site had healed completely.

**Table 1:** Rehabilitation program.

Post HT day	Exercise type	Intensity	Duration of 1 session
0–14 days	Chest physiotherapy (mechanical insufflation-exsufflation, incentive spirometry, and cough splinting)	11–13 of RPE	
14–21 days	Aerobic exercise (portable half-automated ergometer, portable hand-held ergometer)	11–13 of RPE	10 min
	Resistance exercise (elastic band)		
	Incentive spirometry		
21–28 days	Aerobic exercise (portable hand-held ergometer)	11–13 of RPE	25 min
	Resistance exercise (tilt-table standing)		
	Incentive spirometry		
>28 days	Aerobic exercise + resistance exercise (walking and training with aid)	11–13 of RPE	30 min

Three years postoperatively, he became a wheelchair user, with decreased motor power of trace grade in his hip adductors and knee extensors. His weight increased to 99 kg due to treatment with steroids and immunosuppressants, and his activity decreased due to LGMD. Cardiopulmonary exercise testing was performed with an ergometer using the upper extremities, demonstrating a maximal O_2_ consumption of 9.08 mL/kg/min, maximal metabolic equivalent of 2.6, and predicted value of 31%.

## 3. Patient consent

Informed consent was obtained from the patient for the publication of case details and images.

## 4. Discussion

In patients with MD, cardiomyopathy usually starts before the age of 20 years.^[1]^ As dilated cardiomyopathy develops in most patients with MD, leading to advanced heart failure, LVADs and HT are often required. Palladino et al^[2]^ suggested that the long-term clinical outcomes of HT in selected patients with MD are similar to those of a matched cohort of patients who underwent transplantation for idiopathic dilated cardiomyopathy. The group comprised 59 patients with MD, including 2 with LGMD. There are many different types of MD; among them, patients with LGMD are mostly disabled by the age of 20 years. However, many patients have a normal life expectancy. In addition, the symptoms of heart failure in ambulatory patients are more pronounced and severe than those in patients who cannot walk. Accordingly, it is important to maintain one’s function to the best extent possible through appropriate rehabilitation, especially during the postoperative period. However, there are few studies or guidelines for CR programs in patients with LGMD.

Patients receiving HT experience improvements in maximal oxygen consumption, which remains for up to 10 years postoperatively.^[3]^ However, they have a significant decrease in exercise capacity compared with healthy adults. CR is known to improve exercise capacity in the short term and is also safe for patients who have undergone HT, with a 29% reduction during 1-year hospitalization.^[4,5]^ Moreover, one study showed that only 1 week of bed rest reduces skeletal muscle mass,^[6]^ and muscle loss due to prolonged immobilization after surgery can be a barrier to postoperative recovery. Therefore, early CR should be performed after HT.

CR after HT has several factors that distinguish it from CR in other cardiovascular diseases. First, the transplanted heart is denervated by the autonomic nervous system.^[7,8]^ Therefore, patients undergoing HT demonstrate higher resting HR and reduced chronotropism. Similarly, the patient exhibited a blunt HR response during aerobic exercise. Thus, the RPE scale was used instead of the HR to monitor the response of the patient’s heart to exercise to adjust the exercise intensity appropriately. Second, following HT via median sternotomy, patients are routinely advised to comply with upper limb restrictions to prevent the development of sternal complications for 6 weeks. In some guidelines, patients are recommended to not lift >10 pounds of weight and avoid shoulder flexion and abduction >90°.^[9,10]^ Thus, the main exercises are focused on the lower extremities.

Several aspects of patients with LGMD should be considered. As the patient could not walk without a walking aid, walking exercises were not possible. To overcome these limitations, we started the lower extremity exercise using a portable half-automated ergometer in the supine position on the bed for aerobic exercise, until 3 weeks after the surgery. Upper extremity exercises were performed using a portable hand-held ergometer with the upper arms held as close to the trunk as possible. The skeletal muscles of patients with MD are vulnerable to physical stress, and it is important to avoid muscle fatigue. To perform low-intensity exercises to prevent muscle damage, the RPE scale was monitored to ensure that the patient remained between the scores of 11 and 13 with a blunt HR response. There were no changes in serum creatinine kinase and myoglobin levels, and muscle fatigue after exercise was not self-reported.

Although some cases of early CR after HT exist, the advantages of early CR after HT in patients with MD remain undetermined, because patients with MD undergoing HT are rare.^[5]^ However, in a study of 9 cases of MD with HT, the peak oxygen consumption increased after CR and ambulatory function was recovered.^[11]^ Compared to the physical function before HT, the patient also benefited from early CR, ambulatory function recovered with a walking aid, and CR was performed safely. To the best of our knowledge, there are no case reports of detailed CR programs in patients with LGMD who underwent HT. As we could not apply the usual CR program, we attempted to identify a suitable exercise regimen for the patient, create a protocol, monitor his vital signs and laboratory results, and perform rehabilitation. It is significant that the patient’s previous walking ability was maintained even though he was hospitalized at 4 weeks of bed rest.

A limitation of this case is that the patient could not be examined using objective tests. We examined his muscle power using subjective manual muscle testing and not using a dynamometer, isokinetic machine, or surface electromyography. Muscle mass was not examined by computed tomography, bioelectrical impedance analysis, or dual-energy X-ray absorptiometry. Exercise capacity was not measured using cardiopulmonary exercise testing before and after HT, which could reflect the patient’s improvement. Further studies using objective tests are required to assess the effectiveness of early CR for HT in patients with MD. Another limitation is related to the sternotomy precautions. The number of patients with LGMD undergoing surgery through sternotomy, such as those with HT or LVADs, will gradually increase owing to the progression of cardiomyopathy. As sternotomy precautions are recommended by some experts, if qualitative/quantitative evaluation of bone healing becomes possible, these precautions could be effectively tailored for individual patients.

Since patients with LGMD are prone to cardiomyopathy and have a longer life expectancy than other patients with MD, a detailed rehabilitation program that considers the patient’s abilities is necessary. A long-term prospective study is needed to confirm the long-term effects of early CR after HT in patients with MD.

## Author contributions

Conceptualization: Byeong-Ju Lee, Youngmo Kim.

Data curation: Byeong-Ju Lee, Hye Won Lee, Min Kyung Park, Myung-Jun Shin, Ra Yu Yun, Youngmo Kim.

Formal analysis: Byeong-Ju Lee.

Investigation: Byeong-Ju Lee, Youngmo Kim.

Supervision: Byeong-Ju Lee, Myung-Jun Shin, Yong Beom Shin.

Writing – original draft: Youngmo Kim.

Writing – review & editing: Byeong-Ju Lee, Youngmo Kim.
